# Ambulances for London

**Published:** 1904-03-05

**Authors:** 


### The Hospital.

A Journal of

The Medical Sciences and Hospital Administration.

Vol. XXXV.—No. 910. MARCH 5, 1904.

### Ambulances for London.

Few endeavours could be more worthy of support than that for the establishment of a sufficient horse or motor ambulance system for the whole of the metropolis, as advocated by Mr. Reginald Harrison, F.R.C.S., who published a pamphlet on the subject which has been widely circulated. It is based upon his experience at Liverpool, where he had frequent opportunities of seeing the difficulties which were encountered in the conveyance of sick or injured persons. He has also inspected the methods adopted in the United States. The particulars issued by the Hospitals Association which, thanks to Mr. H. L. Bischoffsheim's munificence, has had under its control the street ambulance system of the metropolis as given on another page of this journal to-day, show that the circumstances and needs of London are not likely to be properly met by an expensive system of horse or motor ambulances. The work already accomplished has simplified the solution of the ambulance problem in London, and the actual initial cost and yearly outlay upon a complete system which shall supplement to the required extent the present Bischoffsheim street ambulance organisation need not be very material. It is essential, however, that the County Council, which has control of the streets and sites within the County of London shall now step in and by offering facilities to secure or by providing sites and accommodation enable the Bischoffsheim street ambulance system to be perfected. By such a scheme London can be ambulanced upon a plan based upon experience and knowledge, which will greatly reduce the expenditure and secure the maximum of efficiency with the least possible delay.

With the view to secure a complete ambulance system for London the Hospitals Association and a new body calling itself the Metropolitan Ambulance Association, of which Mr. Harrison is president, have invited the candidates at the County Council elections of the 5th of March, whether they call themselves Conservatives or Progressives, to pledge themselves, if elected, to move the new Council to undertake this work. The bones of a Progressive are as fragile as those of his political opponents; and the sharp extremities of their broken ends would be as likely to convert a simple fracture into a compound one in the course of transit in a four-wheeled cab between a hospital and the scene of a street accident. The "Progressives" may also fairly be called upon to remember that the numerical preponderance of the working man, together with the nature of his occupations, renders him far more liable to accidental injury than any other class of the community, and hence that, by accepting responsibility for his conveyance to hospital in time of need, they will be acting in complete accordance with their reiterated professions of interest in his welfare, as well as with the undoubted fact of their interest in his vote.

The best scheme of ambulance service would imply the establishment of easy telephonic communication between every portion of London and the nearest ambulance station, and the despatch, in prompt answer to a call, of a vehicle fitted with stretcher, and supplemented by a capable first-aid attendant, who should be provided with splints, bandages, restoratives, means of arresting hæmorrhage, and all other likely requirements. In some of the towns in which such an arrangement exists, there is, we believe, a charge made for the use of the ambulance to those who are supposed to be able to pay; but in London, if the establishment were under the control of the County Council, it is manifest that every ratepayer would have an equal claim upon its services, and that no payment could properly be demanded. The expense, apart from the first equipment, would not be very large. Unfortunately, the whole project is one of which the full realisation is dependent upon Parliament, and the business of Parliament is dependent upon conditions which it is impossible to foresee. Only last week the House of Commons devoted four hours and a half, and a single member spoke for two hours, on a Bill for the protection of a few music publishers against piracy; but it may be a long time before a similar period could be commanded for the protection of the lives and limbs of the thousands of people who annually suffer in the streets of London from accident or illness, and whose numbers are growing year by year with the growing amount and complexity of the traffic. We have no access to the most recent statistics; but the recorded street accidents in the metropolitan area, which were 4,766 in 1891, had risen to 7,995 in 1901, and are practically certain to go on increasing. The inevitable substitution of motor for horse drawn vehicles will probably diminish them to some extent; but before many years have passed the streets will probably be as much too small for the motor traffic as they now are for that which they are at present called upon to receive. The demand is becoming urgent, and the neglect of it in London, in the face of the manner in which it is supplied elsewhere, is a blot at once upon our municipal government and upon civilisation. We can but trust that Mr. Bischoffsheim and his fellow-workers may succeed in convincing the members of the new Council of the duty of interference; and that they in turn may be able for a moment to divert the attention of politicians to the accomplishment of a good and useful piece of work.

